# Gpr97 is dispensable for metabolic syndrome but is involved in macrophage inflammation in high-fat diet-induced obesity in mice

**DOI:** 10.1038/srep24649

**Published:** 2016-04-19

**Authors:** Jueping Shi, Xiaoyu Zhang, Shaoying Wang, Jinjin Wang, Bing Du, Zhugang Wang, Mingyao Liu, Wenzheng Jiang, Min Qian, Hua Ren

**Affiliations:** 1 Shanghai Key Laboratory of Regulatory Biology, Institute of Biomedical Sciences and School of Life Sciences, East China Normal University, Shanghai, China; 2 Shanghai Research Center for Model Organisms, Shanghai, China

## Abstract

Local inflammation in tissues is one of primary causes in development of metabolic disorder in obesity. The accumulation of macrophages in some tissues can induce inflammatory reactions in obesity. Gpr97 is highly expressed in some immunocytes, but its potential role in inflammatory regulation has not been revealed clearly. In our research, we investigated Gpr97 in regulating macrophage inflammation and metabolic dysfunction in the high-fat diet (HFD)-induced obese mice. The major metabolic phenotyping were not different after *Gpr97* knockout in HFD-fed mice. Similar pathological alterations in adipose tissue, liver, and kidney were observed in *Gpr97*^−/−^ HFD mice compared with WT-HFD mice. In white adipose tissue, loss of Gpr97 reduced the ratio of M1-macrophages and increased the M2-macrophage ratio, which was opposite to that seen in the wild-type HFD mice. More macrophages invaded in the liver and kidney after Gpr97 knockout in HFD mice. Furthermore, the levels of TNF-α were higher in the liver and kidney of *Gpr97*^−/−^ HFD mice compared to those in wild-type HFD mice. The data indicate that Gpr97 might be required for local inflammation development in obesity-relative tissues, but does not play a role in metabolic disorder in HFD-induced obesity.

In recent decades, the rate of obesity has increased, especially in Europe and other developed countries^1^,^2^. According to WHO statistics, the worldwide prevalence of obesity nearly doubled between 1980 and 2008, and obesity has become a serious public health problem^3^. Obesity is recognized as a severe metabolic disorder and is likely to be a risk factor for type 2 diabetes, fatty liver disease, chronic kidney diseases, and some cancers^4^,^5^,^6^,^7^. However, there is evidence that obesity is also a kind of low-grade chronic inflammatory disease, and there is a clear link between tissue inflammatory and metabolic responses in obesity^8^,^9^. Obese adipose tissue secretes more inflammatory cytokines, including TNF-α and IL-6, compared with lean tissue^10^,^11^. As in other inflammatory responses, obesity causes an increase in the infiltration of immune cells including macrophages, into key metabolic tissues, which would then be involved in the pathogenesis of metabolic syndrome^12^. In adipose tissue, liver, skeletal muscle, and kidney, macrophage-associated local inflammatory response contributes to the development of obese metabolic dysfunction, such as insulin resistance and carbohydrate accumulation^9^,^13^. Furthermore, it is generally thought that obesity affects the balance of the M1/M2 macrophage ratio in these tissues. Furthermore, the imbalance of active macrophage phenotypes plays an important role in the pathogenesis of metabolic disorders. In adipose tissue, a high-fat diet (HFD) induces to enhance the population of M1-macrophages, which promotes the concentration of pro-inflammatory cytokines, while M2 anti-inflammatory macrophages are down-regulated^8^,^14^. During the process of HFD-induced obesity, the secreted inflammatory factors will attract multiple immune cells that will participate in this process, including T cells, B cells, and mast cells. Then various activate inflammatory pathways were detected to find out the relationship between immune system and metabolic syndrome^9^.

Cytokines, immune cell activation, and surface markers of immune cells have been key factors in studies of immune cell-mediated inflammation in HFD-induced metabolic dysfunction. Several G-protein-coupled receptors (GPCRs) have been identified as important regulators in obese metabolic disorder, such as GPR119 and GPR120^15^,^16^. GPR97, as well as GPR56 and GPR114, is a member of the adhesion GPCR family, whose encoding genes are all on the long arm of chromosome 16^17^. In mice, Gpr97 is only expressed in the hypothalamus, heart, kidney, and especially highly on thymus, spleen and blood^18^. In immune cells, GPR97 is expressed on leukocytes, such as neutrophils, eosinophils, and mast cells^17^. Recently, it was reported that the GPR97 plays a role in regulating B-cell fate^19^ and migration of lymphatic endothelial cells^20^. It was also reported that the expression of GPR97 was significantly down-regulated when using colony-stimulating factor-1 (CSF-1/M-CSF) or granulocyte macrophage colony-stimulating factor (GM-CSF) to stimulate primary human macrophages differentiation from monocytes^21^, but its molecular mechanism in macrophage polarization and the related inflammatory response have not yet been reported. It seems that GPR97 might play an important role in immune system, but whether it also functions in inflammation during obesity has yet to be determined. Here we used *Gpr97*-knockout mice to elucidate whether Gpr97 has a role in macrophage-related inflammation and obesity-induced metabolic syndrome.

## Results

### Gpr97 is not involved in obesity and fasting blood glucose in HFD mice

**Figure 1:**
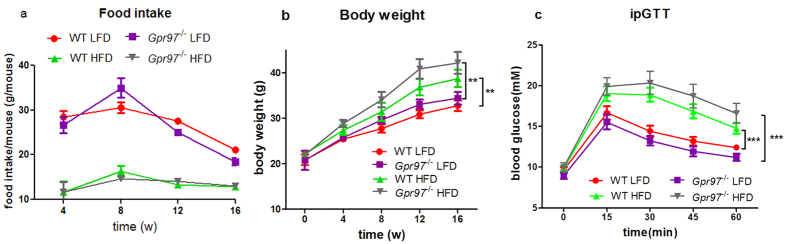
The obese phenotype and fasting blood glucose of high-fat diet (HFD)-induced mice. (**a**) Food intake. The average grams food intake in WT and *Gpr97*^−/−^ mice each month. (n = 8) (**b**) Body weight. The growth of body weight in WT and *Gpr97*^−/−^ mice by chow or HFD feeding. (n = 6–8) (**c**) Intraperitoneal glucose tolerance test (ipGTT). The levels of blood glucose were detected at 0, 15, 30, 45 and 60 minutes after intraperitoneal inject in chow or HFD fed mice (n = 8). Data are shown as means ± s.e.m. **0.01 < P < 0.05, ***P < 0.01.

The mice were fed with 60% calories in fat for 16 weeks to induce the metabolic syndrome. Metabolic phenotype, including food intake, body weight and glucose tolerance, was examined in mice. The quantity of food intake and body weight was recorded once a month. According to the collected data, there was no obvious difference in food intake between WT and *Gpr97*^−/−^ HFD-fed mice (Fig. 1a). Although HFD-fed mice were significantly heavier than chow-fed mice, especially after 12-weeks (P < 0.05), no alteration was shown in *Gpr97*-deficient HFD-fed mice (Fig. 1b). Moreover, we used an intraperitoneal glucose tolerance test (ipGTT) to examine glucose tolerance in our mice model. The fasting blood glucose of HFD mice was much higher than that of the chow-fed mice from 15 min (P < 0.01), but there was no change after loss of *Gpr97* in HFD mice compared with WT HFD mice (Fig. 1c). These data indicate that Gpr97 deficiency does not influence the body weight or glucose tolerance of HFD mice.

### Gpr97 does not play a role in HFD-induced metabolic syndrome

**Figure 2:**
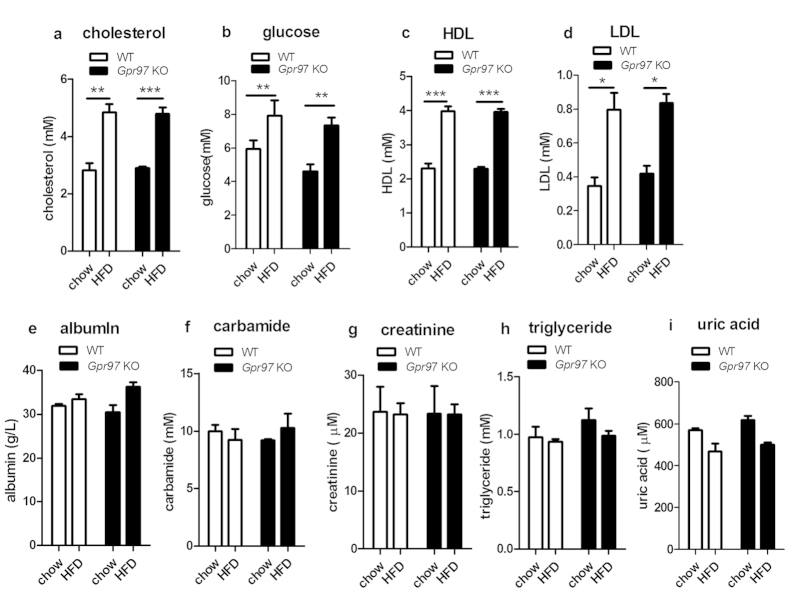
Analysis of major metabolites in serum in mice. (**a**) cholesterol, (**b**) glucose, (**c**) high-density lipoprotein (HDL), (**d**) low-density lipoprotein (LDL), (**e**) albumin, (**f**) carbamide, (**g**) creatinine, (**h**) triglyceride, (**i**) uric acid. Data are shown as means ± s.e.m. (n = 8). *P < 0.05, ** 0.01 < P < 0.05, ***P < 0.01.

To determine whether *Gpr97* impacts on HFD-induced metabolic syndrome, the levels of some metabolites in serum were measured in mice. The levels of cholesterol, glucose, high-density cholesterol lipoprotein (HDL), and low-density cholesterol lipoprotein (LDL) in serum were increased in both HFD-fed WT and *Gpr97*^−/−^ mice. However, there was no significant alteration of these serum parameters after *Gpr97* deficiency in HFD-fed mice (Fig. 2a–d). The levels of albumin, carbamide, creatinine, triglyceride, and uric acid in serum were measured in mice. However, the HFD did not induce these indicators altering even in *Gpr97*^−/−^ HFD mice (Fig. 2e–i). According to the metabolite analysis, Gpr97 is not involved in metabolic syndrome during HFD-induced obesity in mice.

### Gpr97 is involved in the M1/M2 polarization of macrophages in white adipose tissue

**Figure 3:**
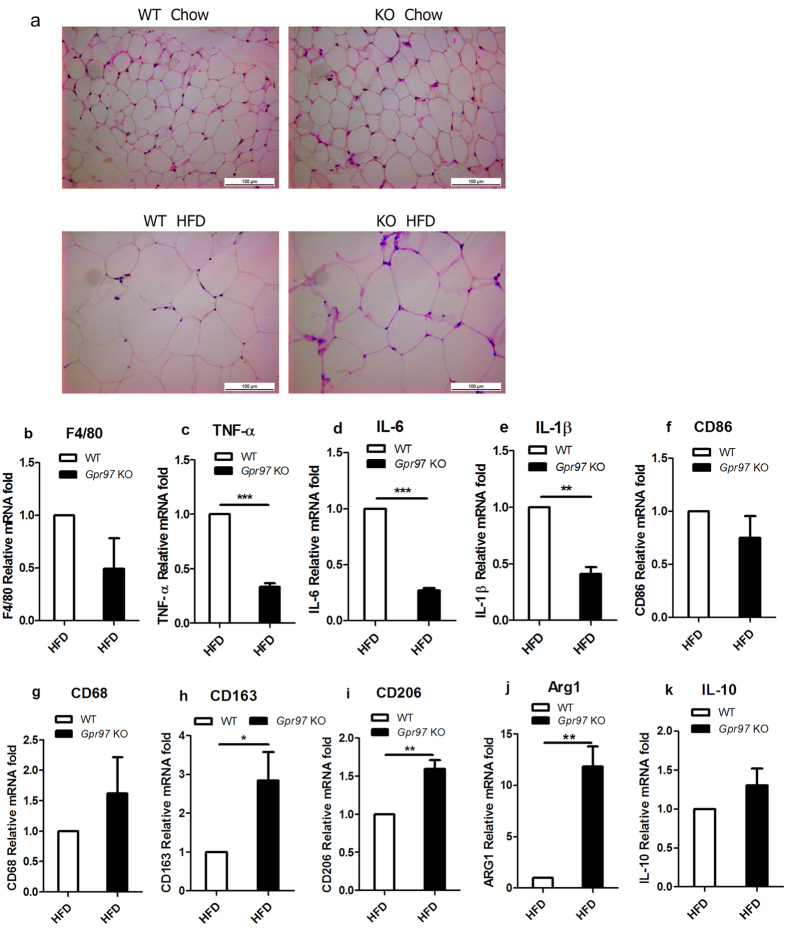
Detection of macrophages polarization in white adipose tissue (WAT) in HFD mice. (**a**) Hematoxylin and eosin staining of WAT sections of WT and *Gpr97*^−/−^ mice. Pictures were captured at 40× under optical microscope. (**b**) The expression of the relative factor associated with total macrophages F4/80 in mRNA levels in WAT. (**c**–**f**) The expression of relative factors in mRNA levels associated with M1-polarized macrophages in WAT. (**g**–**k**) The expression of relative factors associated with M2-polarized macrophages in WAT. Data are shown as means ± s.e.m. (n = 8). 0.01 < P < 0.05, ***P < 0.01.

White adipose tissue (WAT), which is a critical regulator of adiposity and energy metabolism, has been found to undergo metabolic changes during a high-fat diet^22^. Adipocyte hypertrophy and hyperplasia occurs in HFD-induced obesity, including overgrowth of WAT^23^. In our results, hypertrophy of WAT was a typical change in HFD mice, but the morphology of WAT did not significantly alter between WT and *Gpr97*^−/−^ HFD mice (Fig. 3a). It has been reported that the HFD will result in the accumulation of macrophage in WAT, which can lead to inflammation^24^. According to the alteration in the mRNA level of the macrophage marker F4/80, we found that Gpr97 deficiency can cause a reduction in macrophage invasion of WAT in HFD-fed mice (Fig. 3b). The general view of the inflammatory reactions in WAT is an imbalance in the ratio of M1/M2 macrophages^24^. We analyzed the relative mRNA expression of cytokines associated with M1- and M2-polarized macrophages to confirm the function of Gpr97 in M1/M2 polarization of WAT macrophages. The M1-polarized-related parameters TNF-α, IL-6, IL-1β and CD86 displayed a decrease in *Gpr97*^−/−^ HFD mice compared to WT HFD mice (Fig. 3c–f). Meanwhile, the M2-polarized-related parameters, including CD68, CD163, CD206, Arg1 and IL-10 were increased in *Gpr97*^−/−^ HFD mice (Fig. 3g–k). The accumulation of M1 “pro-inflammatory” macrophages and the reduction of M2 “anti-inflammatory” macrophages in white adipose tissue can lead to adipose tissue dysfunction^24^. We found that *Gpr97* deficiency can reverse this imbalance of M1/M2 macrophages in WAT. In *Gpr97*^−/−^ HFD mice, M2 macrophages were increased and M1 macrophages were decreased obviously, which indicate that Gpr97 might contribute to the local inflammatory reactions charged of M1-macrophages. In the obesity induced by HFD, loss of Gpr97 will cause opposite imbalance of M1/M2 and reduce levels of inflammatory cytokines in WAT of mice.

### Gpr97 enhances hepatic inflammation in HFD mice

**Figure 4:**
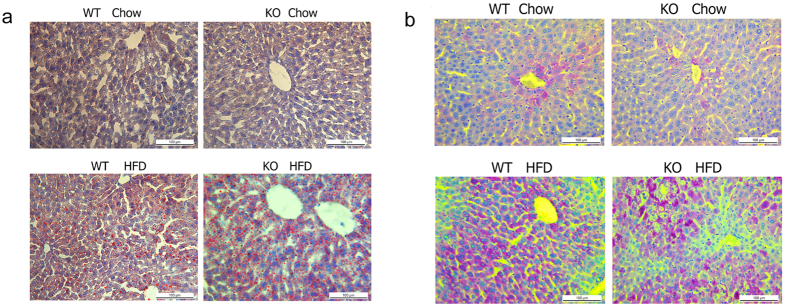
Microphotographs of the hepatic sections of control mice and *Gpr97*^−/−^ mice. (**a**) The Oil-red staining of liver sections of WT and *Gpr97*^−/−^ mice. (**b**) The PAS staining of liver sections of WT and *Gpr97*^−/−^ mice. The lung sections were observed at 10× magnification under optical microscope.

The liver plays an important role in lipid metabolism, and hepatic steatosis was measured in an obese animal model^8^. HFD not only introduced obesity, but was also involved in glycometabolism and lipid metabolism in the liver. In our HFD mice, we used the Oil Red O stain to visualize hepatic neutral lipid accumulation in liver sections. The accumulation of lipid was severe in the livers of mice fed the HFD, but this accumulation was not significantly different between WT and *Gpr97*^−/−^ HFD mice (Fig. 4a). Next, we measured the metabolism of carbohydrate in the liver by periodic acid-Schiff (PAS) staining, and found that there was more glycogen storage in the liver in HFD-fed mice. However, there was no difference between WT and *Gpr97*^−/−^ mice (Fig. 4b). The loss of Gpr97, therefore, did not affect lipid or glycogen storage, or metabolic disorder, in the HFD-induced liver.

**Figure 5:**
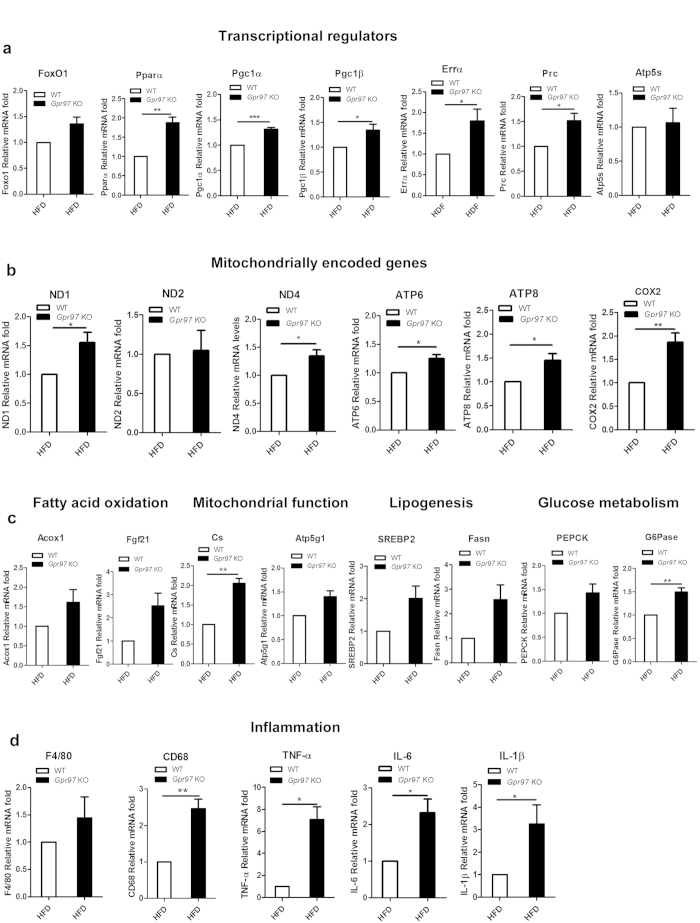
Analysis of metabolite, transcriptional, inflammatory factors in mRNA levels in the livers of HFD-fed mice. The expression of genes in mRNA levels which are associated with transcriptional regulators (**a**), mitochondrially encoded genes (**b**), fatty acid oxidation, hepatic mitochondrial function, lipogenesis, glucose metabolism (**c**) and inflammatory factors (**d**) in HFD mice. Data are shown as means ± s.e.m. (n = 8). *P < 0.05, ** 0.01 < *P* < 0.05.

To determine the function of Gpr97 in the liver of HFD mice, we measured the mRNA expression of metabolic factors, including those involved in transcriptional regulators which influence pathways of fatty acid oxidation (FoxO1, Pparα, Pgc1α, Pgc1β, Errα, Prc and Atp5s) (Fig. 5a), mitochondrially encoded genes (ND1, ND2, ND4, ATP6, ATP8 and COX2) (Fig. 5b), fatty acid oxidation (Acox1, Fgf21), mitochondrial function (Cs, Atp5g1), lipogenesis (SREBP2, Fasn), glucose metabolism (PEPCK, G6Pase) (Fig. 5c), and inflammation (F4/80, CD68, TNF-α, IL-6 and IL-1β) (Fig. 5d) 25. These genes, which are involved in the major metabolic pathways and inflammatory reactions in the liver, showed slightly higher expression in *Gpr97*^−/−^ mice than in WT mice under a HFD-fed. Gpr97 deficiency, therefore, will aggravate some key gene expression which are involved in hepatic metabolism such as transcriptional regulation in fatty acid oxidation and mitochondrial function. Moreover, the inflammatory factor TNF-α, IL-6 and IL-1β were increased after *Gpr97* knockout in HFD-fed mice. The higher expression of F4/80 and CD68 indicates that more macrophages gathered in the liver of *Gpr97*^−/−^ HFD mice, and that the loss of Gpr97 may cause more serious inflammation and metabolic stress in the liver of obese mice.

### Effect of Gpr97 on HFD-induced renal inflammation

**Figure 6:**
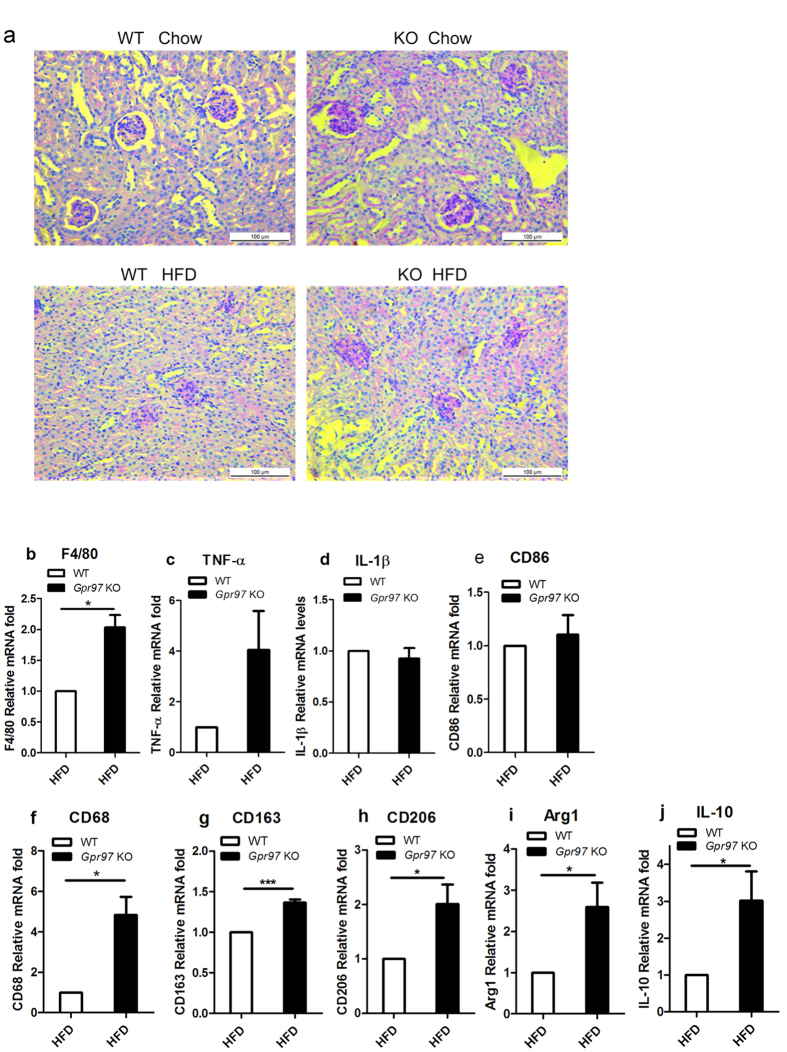
The HFD-induced inflammation analysis in kidney in the WT and *Gpr97*^−/−^ mice. (**a**) The morphological evaluation of kidney by PAS staining after HFD fed in mice. (**b**) Detecting of macrophage makers F4/80 in mRNA level to determine invasion of macrophage into kidney in HFD mice. (**c**–**e**) The expression of relative markers in mRNA levels associated with M1-polarized macrophages in kidney of HFD-fed mice. (**f**–**j**) The analysis of markers of M2-polarized macrophages in mRNA level in kidney of HFD-fed mice. Data are shown as means ± s.e.m. (n = 8). *P < 0.05.

HFD can also cause renal inflammation and hypofunction^26^. Renal structural changes were evaluated by histochemistry with the PAS stain after HFD. Clear and unbroken structures of renal sections were shown in both WT and *Gpr97*^−/−^ chow-fed mice. There was also no interstitial fibrosis in the renal tubules or glomeruli. Mice fed the HFD exhibited renal injury, such as renal tubular epithelial cell cytoplasm vacuole degeneration, mesangial matrix expansion, and increased renal interstitial cells (Fig. 6a). However, the absence of *Gpr97* did not impact upon the HFD-induced renal injury. In HFD-fed mice, a high inflammation status would increase renal injury and lipid metabolic disorder, and some inflammatory markers, such as TNF-α, IL-6, and MCP-1, were induced by the HFD^27^,^28^. Next, we measured the alterations in renal inflammatory cytokines in HFD mice after *Gpr97* knockout. In *Gpr97*-deficient HFD mice, there were more macrophages invading the kidney according to the increase in F4/80 mRNA levels (Fig. 6b). Meanwhile, the inflammatory factor TNF-α was increased in *Gpr97*^−/−^ HFD mice compared with that in WT HFD mice (Fig. 6c), but there was no significant change in IL-1β and CD86 after *Gpr97* knockout in HFD mice (Fig. 6d,e). So Gpr97 did not affect ratio of M1-polarized macrophages in kidney of HFD-fed mice. Further, we detected markers of M2- polarized macrophages including CD68, CD163, CD206, Arg1 and IL-10 in kidney and found increasing of those in kidney after *Gpr97* deficiency in HFD-mice (Fig. 6f–j). According to our results, Gpr97 might be involved in the renal macrophage-inflammatory reaction and macrophage invasion in HFD mice, but not in the imbalance in the ratio of M1/M2 macrophages.

## Discussion

Obesity is a chronic low-grade inflammatory disease^29^. Inflammation plays an important role in the pathological process of obesity. For obese rodents and humans, activated macrophages infiltrate metabolic tissues, including adipose tissue^10^,^12^, liver^30^,^31^, and kidney^32^, and such a large number of macrophages can cause serious local inflammation. Furthermore, they can impair the balance of the resident macrophage population and can affect the metabolic regulation function in these tissues^9^,^33^. However, how specific genes involved in the regulation of tissue-specific inflammation and metabolic function are induced by a HFD has rarely been reported. A family of GPCRs were found that have important roles in various physiological and pathological processes, such as inflammatory regulation^34^,^35^. The potential functions of Gpr97, which is an adhesion GPCR, are little known. Gpr97 has shown effects on B-lymphocyte development^19^ and migration of lymphatic endothelial cells^20^. Here we focused on the role of Gpr97 on tissue-specific inflammation in HFD-induced obese mice.

Firstly, we determined whether Gpr97 had a function in HFD-induced metabolic abnormalities. The general physiological parameters, the intraperitoneal glucose tolerance, and metabolic characterization were examined in both WT and *Gpr97*^−/−^ mice fed a chow diet or the HFD. Our results showed that the *Gpr97*^−/−^ mice had almost equal levels of physiological parameters and serum parameters after the HFD. In the ipGTT test, there was also no significant difference between the wild-type and *Gpr97*-knockout obese mice (Figs 1 and 2). Therefore, Gpr97 might not protect from or sensitize to the development of HFD-induced obesity, glucose tolerance, and systemic metabolic regulation in mice. Next, we focused on the function of Gpr97 in some important tissues involved in HFD-induced obesity.

WAT is regarded as an active metabolic tissue that can produce adipokines, as well as pro- or anti-inflammatory cytokines capable of regulating carbohydrate, lipid metabolism and local inflammation^36^. To respond to excess nutrients, WAT showed hypertrophy and hyperplasia in HFD-fed mice (Fig. 3a). It was reported that adipocyte size is related to insulin sensitivity and human with smaller adipocytes have a lower degree of inflammation^37^. Moreover, obesity can also induce macrophage invasion into WAT and can lead to WAT inflammation. The accumulated macrophages in WAT secrete pro-inflammatory factors to enhance metabolic dysfunctions^38^. Our results indicate that *Gpr97* deficiency reduces macrophage invasion into WAT (Fig. 3b). The balance in the ratio of the macrophage population in WAT plays a key role in the development of HFD-induced obesity. In lean rodents, there are more M2-macrophges in adipose tissue, which are known to secrete anti-inflammatory cytokines^39^. In contrast, in obese animals, more activated macrophage and monocytes were recruited to the WAT, which turn into M1-macrophages. During this process, pro-inflammatory cytokines, including TNF-α and IL-1β, produced by M1-macrophages are capable of limiting glucose uptake in peripheral tissue^36^,^40^,^41^. Interestingly, we found that less macrophages invasion in the WAT and there was a reversed imbalance of ratio of M1/M2 macrophages in the WAT of obese *Gpr97*-knockout HFD-fed mice. This indicates that loss of Gpr97 will cause an M2-macrophage-related anti-inflammatory response to modulate healing repair process in WAT. Such evidence suggests that Gpr97 could modulate macrophage polarization during metabolic disorder and local inflammation in adipose tissue. However, further studies should be carried out to determine the potential mechanisms for macrophage polarization and the inflammatory reaction regulated by Gpr97 in the HFD-induced WAT.

Furthermore, for metabolic organs, the liver has a central position in the endocrine and metabolic system. For example, it plays a vital role in the metabolism of sugar, sugar storage and decomposition, and regulation of blood glucose. Excessive lipogenesis contributes to the development of hyperglycemia and hepatic steatosis^9^,^42^. Lipid and glycogen accumulation occurred after 16 weeks of the HFD in both WT and *Gpr97*-knockout mice. Similar to previous results in white adipose tissue, Gpr97 may not be important enough to affect the hepatic phenotypic alterations seen during HFD-induced obesity. However, *Gpr97* deficiency led to a slight increase in the expression of major metabolic genes involved in different physiological process of the liver. It was known that HFD causes hepatic lipid accumulation and that the complex and coordinated metabolic processes are also aggravated, including lipogenesis, fatty acid oxidation, glucose metabolism, mitochondrial function and metabolic transcriptional regulation in the liver^25^,^43^,^44^,^45^,^46^. According to our results, loss of *Gpr97* might slightly increase these metabolic programs in mRNA levels. Meanwhile, inflammatory cytokines including TNF-α, IL-6 and IL-1β and number of macrophages were increased sharply in the liver after *Gpr97* knockout in HFD mice. In summary, Gpr97 may participate in the regulation of expression of metabolic factors and the macrophage-related inflammatory response in liver, and the loss of Gpr97 may cause more serious hepatic dysfunction and inflammation. But how Gpr97 regulates altering of these factors in HFD-induced obesity should be further investigated.

Moreover, obesity is a potential risk factor for kidney disease in which fibrosis and inflammation play important roles. In HFD-fed mice, renal interstitial fibrosis and inflammatory cells were increased, which may develop into diabetes even diabetic nephropathy^26^. Whether Gpr97 contributes to the process is being considered. Our results indicate that Gpr97 did not affect the structural changes seen in the kidney during a HFD in mice (Fig. 6a). However, Gpr97 seems to play an underlying role in the regulation of macrophage infiltration in HFD-induced renal inflammation. It was reported that macrophages can also be activated by a HFD and can infiltrated into the kidney^47^. After the loss of *Gpr97*, more macrophages invaded into the kidney, and the pro-inflammatory factor TNF-α was slightly increased in HFD-fed mice. Then we detected the altering of ratio of M1 and M2-related macrophages in kidney. It was found that more number of M2 macrophages and no change of ratio of M1 macrophages in kidney after Gpr97 knockout in HFD-fed mice. Therefore, we propose that Gpr97 is also involved in macrophage-related inflammation but not involved in macrophage polarization in kidney. How Gpr97 affects renal macrophage-related inflammatory response should be investigated further to reveal the potential regulatory mechanisms of Gpr97 in HFD-induced obesity.

In summary, we report the potential role of Gpr97 in metabolic syndrome and inflammation in HFD-fed mice. No phenotypic change was observed between wild-type and *Gpr97*^−/−^ HFD-fed mice, which indicates that Gpr97 does not play a role in the phenotypic development and metabolic homeostasis under such extreme conditions. However, further studies demonstrated that the absence of *Gpr97* could recruit more macrophages into metabolic organs, including the liver and kidney. Then it would lead to initiation of tissue-specific macrophage-inflammation and promotion of metabolic regulation. Further, there was consistent evidence to suggest that Gpr97 was involved in macrophage polarization in WAT in HFD-induced obesity in mice. However, more evidence should be found to explain the mechanism of inflammation regulated by Gpr97 in the process of obesity induced by HFD.

## Methods

### Animals and diet

Six-week-old male C57BL/6 mice (Shanghai Laboratory Animal Company, China) and *Gpr97*^−/−^ C57BL/6 mice were housed in the Laboratory Animal Center of East China Normal University. *Gpr97*^−/−^ mice were donated by the Dr. Zhugang Wang Laboratory (Shanghai Research Center for Model Organisms, Shanghai, China). Mice were raised in a regulated temperature (22 ± 2 °C) with a 12-h light/12-h dark cycle, and free access to food and water. Animals were fed a standard chow diet or a high-fat diet for 16 weeks. The blood glucose concentration, body weight, food intake, and water intake were measured once a week to monitor the general changes during the whole feeding process. Before experiments, the overnight fasted mice were sacrificed by dislocation of the vertebrae to obtain tissues and blood samples. Liver and kidney tissues were weighed and kept at −80 °C for subsequent analysis. All animal experiments conformed to the regulations drafted by the Association for Assessment and Accreditation of Laboratory Animal Care in Shanghai and were approved by the East China Normal University Center for Animal Research.

### Intraperitoneal glucose tolerance test

At the end of the feeding phase, an intraperitoneal glucose tolerance test (ipGTT) was performed on mice that had been fasted for 12 hours. Mice were intraperitoneally injected with glucose (1 g/kg), and then blood glucose concentration from vein blood were measured at 0, 15, 30, 45, and 60 minutes using ACCU-CHEK Active (Roche, Switzerland).

### Blood biochemical indicator detection

Mice were fed with the high-fat diet or chow diet for 16 weeks to detect blood biochemical indicators. Samples were collected from the 16–18 h fasted mice after sacrifice, and then serum was obtained by centrifuging at 3000 g for 10 min at 4 °C. The serum was used to determine the levels of some major biochemical indicators, such as triglyceride (TG), cholesterol (CHO), and the serum lipid profile. The test of blood metabolites was conducted by ADICON Clinical Laboratory, Inc. (Shanghai, China).

### Tissue staining

Tissues, including epididymal fat tissue, liver, and kidney, from all sacrificed mice were collected and fixed in 4% paraformaldehyde overnight. Samples were incubated in graded ethanol, followed by embedding in paraffin. Six-μm sections, which were obtained by using a slicer (Leica, Germany), were stained with periodic acid-Schiff (PAS) stain for pathological examination^48^. The slices were incubated in periodic acid alcohol before PAS staining, and then were washed with sulfurous acid, followed by nucleolus staining using hematoxylin and eosin and mounting in glycerol.

Alternatively, fixed tissues were dehydrated in gradient sucrose solution, frozen in optimal cutting temperature (OCT) compound (SAKURA Tissue-Tek, USA). Ten-μm sections were obtained with frozen section (Leica, Germany) at −10 °C, and stained with Oil Red O (Sigma, USA) to observe the lipid accumulation.

### RNA isolation and reverse transcription

Total RNA was isolated from murine liver, adipose tissues and kidney using TRIzol reagent (Invitrogen, USA) according to the manufacturer’s instructions. cDNA was reverse transcribed from RNA using the PrimeScript RT Master Mix Kit (TaKaRa, Japan), which was used as the template for the following real-time PCR reaction.

### Real-time PCR

Quantitative real-time PCR was performed on a Stratagene Mx3005P Real-time PCR System (Agilent Technologies, USA), using SYBR Premix Ex Taq™ (TaKaRa, Japan) and following the manufacturer’s instructions. Transcript levels of the housekeeping gene β-actin in the each sample were used for normalization. The gene-specific primer sequences are listed in Table 1.

### Statistical analysis

All the experimental data are presented as means ± s.e.m. Statistical significance was assessed using a two-tailed unpaired Student’s t test or one-way analysis of variance (ANOVA) using SPSS 20.0 software (IBM). Statistical significance was considered to be at P < 0.05.

## Additional Information

**How to cite this article**: Shi, J. *et al*. Gpr97 is dispensable for metabolic syndrome but is involved in macrophage inflammation in high-fat diet-induced obesity in mice. *Sci. Rep.* **6**, 24649; 10.1038/srep24649 (2016).

## Figures and Tables

**Table 1:** Sequence-specific primers.

Gene name	Primers (5′- 3′)	Size/bp
forkhead box O1(FoxO1)	SENSE: AAGGATAAGGGCGACAGCAA	121
	ANTISENSE: TCCACCAAGAACTCTTTCCA	
peroxisome proliferator activated receptor α (Pparα)	SENSE: CCTGAACATCGAGTGTCGAATAT	322
	ANTISENSE: GGTTCTTCTTCTGAATCTTGCAGCT	
PPARγ coactivator-1α (Pgc1α)	SENSE: AATGCAGCGGTCTTAGCACT	131
	ANTISENSE: TTGTGGCTTTTGCTGTTGAC	
PPARγ coactivator-1β (Pgc1β)	SENSE: GCCTCTCCAGGCAGGTTCA	66
	ANTISENSE: TAGAGAACTCAGTCCAGAAGGCTTT	
Estrogen-related receptor α (Errα)	SENSE: GCAGGGCAGTGGGAAGCTA	127
	ANTISENSE: CCTCTTGAAGAAGGCTTTGCA	
peroxisome proliferator-activated receptor γ coactivator-related 1 (Prc)	SENSE: CCATCCAGCCCGTCTAAGG	81
	ANTISENSE: ATGAACGCCTGCACCACAT	
ATP synthase subunit s (Atp5s)	SENSE: ATTGATGCCACCGATTCTTGTA	61
	ANTISENSE: GCTCTAGGCCCACCATGTGA	
ND1	SENSE: CCCCTTCGACCTGACAGAAG	69
	ANTISENSE: GGGCCGGCTGCGTATT	
ND2	SENSE: CAAGGGATCCCACTGCACAT	90
	ANTISENSE: GAGTAGCGGGTAGATTTGGATTAAAA	
ND4	SENSE: ATCACTCCTATTCTGCCTAGCAAAC	77
	ANTISENSE: GAAGTCCTCGGGCCATAATTATAGT	
ATP6	SENSE: AATTACAGGCTTCCGACACAAAC	79
	ANTISENSE: TGGAATTAGTGAAATTGGAGTTCCT	
ATP8	SENSE: GCCACAACTAGATACATCAACATGATT	130
	ANTISENSE: GGTTGTTAGTGATTTTGGTGAAGGT	
Cyclooxygenase (COX2)	SENSE: CCTTCCTCCCGTAGCAG	206
	ANTISENSE: ACCCAGGTCCTCGCTTA	
peroxisomal acyl-coenzyme A oxidase 1 (Acox1)	SENSE: GCCCAACTGTGACTTCCATT	113
	ANTISENSE: GGCATGTAACCCGTAGCACT	
fibroblast growth factor 21 (Fgf21)	SENSE: CCTCTAGGTTTCTTTGCCAAC	116
	ANTISENSE: CTGGTACACATTGTAACCGTC	
Cs	SENSE: GGAGCCAAGAACTCATCCTG	108
	ANTISENSE: TCTGGCCTGCTCCTTAGGTA	
ATP synthase lipid-binding protein, mitochondrial (Atp5g1)	SENSE: GCTGCTTGAGAGATGGGTTC	89
	ANTISENSE: AGTTGGTGTGGCTGGATCA	
sterol response element binding protein-2 (SREBP2)	SENSE: CCCTTGACTTCCTTGCTGCA	222
	ANTISENSE: GCGTGAGTGTGGGCGAATC	
fatty acid synthase (Fasn)	SENSE: AGCTTCGGCTGCTGTTGGAAGT	121
	ANTISENSE: TCGGATGCCTCTGAACCACTCACA	
G6Pase	SENSE: CCGGATCTACCTTGCTGCTCACTTT	172
	ANTISENSE: TAGCAGGTAGAATCCAAGCGCGAAAC	
phosphoenolpyruvate carboxykinase (PEPCK)	SENSE: CCACAGCTGCTGCAGAACA	65
	ANTISENSE: GAAGGGTCGCATGGCAAA	
F4/80	SENSE:TGACTCACCTTGTGGTCCTAA	111
	ANTISENSE: CTTCCCAGAATCCAGTCTTTCC	
TNF-α	SENSE:TCCCTTTCACTCACTGGC	353
	ANTISENSE: ACTTGGTGGTTTGCTACG	
IL-6	SENSE:TTCTTGGGACTGATGCTG	379
	ANTISENSE: CTGGCTTTGTCTTTCTTGTT	
IL-1β	SENSE:GCCTCAAAGGAAAGAATC	298
	ANTISENSE: GAAACAGTCCAGCCCATAC	
CD86	SENSE:AGCAAGGTCACCCGAAAC	300
	ANTISENSE:GCAGCATCACAAGGAGGAG	
CD68	SENSE:GGACCCACAACTGTCACTCAT	286
	ANTISENSE: AAGCCCCACTTTAGCTTTACC	
CD163	SENSE:GGCTAGACGAAGTCATCTGCAC	144
	ANTISENSE: CTTCGTTGGTCAGCCTCAGAGA	
CD206	SENSE:CTCTGTTCAGCTATTGGACGC	132
	ANTISENSE: CGGAATTTCTGGGATTCAGCTTC	
Arg1	SENSE:CTCCAAGCCAAAGTCCTTAGAG	185
	ANTISENSE: AGGAGCTGTCATTAGGGACATC	
IL-10	SENSE:ATGCTGCCTGCTCTTACTGACTG	216
	ANTISENSE: CCCAAGTAACCCTTAAAGTCCTGC	
β-actin	SENSE:GTACGCCAACACAGTGCTG	212
	ANTISENSE: CGTCATACTCCTGCTTGCTG	
